# Composition tunable cobalt–nickel and cobalt–iron alloy nanoparticles below 10 nm synthesized using acetonated cobalt carbonyl

**DOI:** 10.1007/s11051-012-0991-5

**Published:** 2012-07-04

**Authors:** Matti M. van Schooneveld, Carlos Campos-Cuerva, Jeroen Pet, Johannes D. Meeldijk, Jos van Rijssel, Andries Meijerink, Ben H. Erné, Frank M. F. de Groot

**Affiliations:** 1 Inorganic Chemistry & Catalysis, Debye Institute for Nanomaterials Science, Utrecht University, Universiteitsweg 99, 3584 CG Utrecht, The Netherlands; 2 Van’t Hoff Laboratory for Physical & Colloid Chemistry, Debye Institute for Nanomaterials Science, Utrecht University, Padualaan 8, 3584 CH Utrecht, The Netherlands; 3 Condensed Matter & Interfaces, Debye Institute for Nanomaterials Science, Utrecht University, P.O. Box 80000, 3508 TA Utrecht, The Netherlands

**Keywords:** Synthesis, Nanoparticles, Cobalt alloy, Carbonyl disproportionation, Acetone

## Abstract

A general organometallic route has been developed to synthesize Co_*x*_Ni_1−*x*_ and Co_*x*_Fe_1−*x*_ alloy nanoparticles with a fully tunable composition and a size of 4–10 nm with high yield. In contrast to previously reported synthesis methods using dicobalt octacarbonyl (Co_2_(CO)_8_), here the cobalt–cobalt bond in the carbonyl complex is first broken with anhydrous acetone. The acetonated compound, in the presence of iron carbonyl or nickel acetylacetonate, is necessary to obtain small composition tunable alloys. This new route and insights will provide guidelines for the wet-chemical synthesis of yet unmade bimetallic alloy nanoparticles.

**Electronic supplementary material:**

The online version of this article (doi:10.1007/s11051-012-0991-5) contains supplementary material, which is available to authorized users.

## Introduction

Two-component alloy nanoparticles based on Fe, Co, and Ni are of great interest in the catalysis of, for example, the Fischer–Tropsch synthesis or the decomposition of cellulose (Cabet et al. 1998; Zhao et al. 2011; Jia and Schuth 2011). More than the single metals, bimetallic mixtures make it possible to tune carbon deposition and carbide formation rates, which are crucial for catalytic activity and lifetime (Cnossen et al. 1994; Pinheiro and Gadelle 2001) or the adsorbate bond dissociation energies as a function of the metal *d*-band center as described by the Newns–Anderson model (Nilsson et al. 2008). With bimetallic nanoparticles, catalytic performance is often also enhanced by their superior sintering resistance (Alloyeau et al. 2010; Cao and Veser 2010). Furthermore, an advantage over, for example, Pt, Pd, or Rh is that 3*d* transition metals are abundant and low priced, and can be used to replace expensive noble metals in catalytic processes (Nørskov et al. 2011; Haynes and Lide 2012).

Ideally, bimetallic catalytic nanoparticles should be prepared with a tunable composition and as small as possible, <10 nm, to maximize their surface-to-volume ratio. Although the preparation of bimetallic nanoparticles has been widely researched (Hyeon 2003; Wang and Li 2011), no general approach has been reported to synthesize Co–Ni or Co–Fe particles <10 nm with a tunable composition. Larger Co_*x*_Fe_1−*x*_ particles in the 10–20 nm range have been prepared by thermal decomposition of organometallic compounds in high-boiling organic solvents, for instance using iron pentacarbonyl (Fe(CO)_5_) and Co(η^3^-C_8_H_13_)(η^4^-C_8_H_12_) or Co(N(SiMe_3_)_2_)_2_ (Desvaux et al. 2005), or iron(III) and cobalt(II) acetylacetonate (Chaubey et al. 2007). Smaller particles of 5–8 nm were synthesized using bimetallic carbonyl clusters that contain both iron and cobalt, but with a fixed elemental composition of FeCo_3_ (Robinson et al. 2009). CoNi particles of 30-nm size with a fixed elemental composition were prepared in triethylene glycol with polyvinylpyrrolidone (Hu et al. 2008), and smaller particles were made through a bio-based approach in apoferritine cavities or supported in polymer films (Abes et al. 2003; Gálvez et al. 2010). Monodisperse 8-nm nanoparticles from cobalt and nickel acetate hydrates were also reported but only with a ratio of Co_40_Ni_60_ (Murray et al. 2001). Besides wet-chemical techniques, physical evaporation methods have been used to prepare Co–Fe nanoparticles, but this too did not lead to particles <10 nm with a tunable composition (Reetz et al. 1995; Li et al. 2001; Wang et al. 2003).

Here, we report a novel organometallic method to synthesize colloidal nanoparticles of Co–Ni and Co–Fe with a fully tunable composition and a size of 4–10 nm. Our method relies on a straightforward and inexpensive pre-treatment of dicobalt octacarbonyl in dry acetone before it is thermally decomposed together with iron carbonyl or nickel acetylacetonate. First, the importance of the acetonation step will be demonstrated. Second, the tunability of nanoparticle alloy composition will be examined. Finally, it will be shown how the crystal structure of Co–Ni and Co–Fe nanoparticles can be controlled through the choice and concentration of surfactant molecules present during synthesis.

## Experimental section

### Materials

Nickel(II) acetylacetonate (Ni(acac)_2_; 95 %), cobalt(III) acetylacetonate (Co(acac)_3_; 99.99 %), trioctylphosphine oxide (TOPO; 99 %), dioctyl ether (99 %), 1,2-dichlorobenzene (anhydrous, 99 %), 2-propanol (anhydrous, 99 %), and cyclohexane (anhydrous, 99.5 %) were purchased from Aldrich. Dicobalt octacarbonyl (Co_2_(CO)_8_; hexane stabilized, 95 %), iron pentacarbonyl (Fe(CO)_5_; 99.5 %), oleic acid (OA; 97 %), acetone (anhydrous, 99.8 %), and toluene (anhydrous, 99.99 %) were obtained from Acros. Benzene (≥99.5 %) was obtained from Fluka. All chemicals were used as received.

### Co_*x*_Ni_1−*x*_ nanoparticle synthesis

Co_*x*_Ni_1−*x*_ particles were made by combining literature recipes for the preparation of pure Co or pure Ni nanoparticles and by adding an acetonation step (Murray et al. 2001; Bao et al. 2009). Pure Co nanoparticles were prepared using a Co:OA:TOPO molar ratio of 12.15:2.38:1 (Bao et al. 2009), whereas pure Ni nanoparticles were prepared using a molar ratio nickel(II) acetate tetrahydrate (Ni(CH_3_COO)_2_·4H_2_O) to OA to tributylphosphine to tributylamine of 4:2:1:8 (Murray et al. 2001). Based on this, the following interpolating formulas were used to calculate reactant amounts for a standard synthesis: [OA] = 0.196[Co] + 0.516[Ni] and [TOPO] = 0.0824[Co] + 0.217[Ni], where [i] is the molar concentration of i. First, Co_2_(CO)_8_ and Ni(acac)_2_ were left to dissolve for 30 min in 3 mL of anhydrous acetone in a nitrogen atmosphere glove box, under occasional stirring of the flask by hand. Next, OA and TOPO were simultaneously added to 12 mL dioctyl ether in an adapted round-bottom synthesis flask (see Fig. S1 in Online Resource 1) inside the glove box, and the solution was subsequently heated to 280 °C in a nitrogen Schlenk line outside the glove box. The metal precursor solution was then injected from airtight vials in the hot ligand-containing solvent. Mixtures were refluxed for 30 min, allowed to cool to room temperature, and transferred back to the glove box before further analysis. No amines were used, because we observed that amines destabilize ε-Co nanoparticles (they act as a hard Lewis base forming a strong Co–NH_2_R bond; see Fig. S2 in Online Resource 1). Synthesis series A1–A4 were made in which the Co-to-Ni metal and/or the metal-to-ligand ((Co + Ni)/(OA + TOPO)) ratios were systematically varied. Essentially, series A1 and A2 keep the amounts of surfactants constant and series A3 and A4 keep the amounts of organometallic precursors constant. Exact amounts of metal precursors and ligands used for all Co_*x*_Ni_1−*x*_ syntheses are given in Table S1 in Online Resource 1. It was verified with duplo syntheses for all syntheses in the manuscript that the results are reproducible.

### Co_*x*_Fe_1−*x*_ nanoparticle synthesis

The same procedure as for the Co_*x*_Ni_1−*x*_ nanoparticles was used, but with Ni(acac)_2_ replaced by Fe(CO)_5_ and with the following formulas to calculate the amounts of OA and TOPO: [OA] = 0.196[Co] + 0.75[Fe] and [TOPO] = 0.0823[Co] + 0[Fe]. This was based on literature Fe:OA ratios of 1:1 and 1:3 (Murray et al. 2001; Farrell et al. 2003), and the absence of TOPO in reported Fe nanoparticle syntheses (Farrell et al. 2003). Synthesis series A5–A7 aimed to study the Co_*x*_Fe_1−*x*_ composition dependency on the organometallic precursors and organic ligands concentrations. Exact amounts of the chemicals used can be found in Table S2 in Online Resource 1.

### Alternating gradient magnetometer (AGM) measurements

A volume of 4 μL of dioctyl ether nanoparticle dispersion was added to airtight glass vials inside the glove box. Magnetization curves were measured using a MicroMag 2900 AGM (Princeton Measurements Corporation). Volume-averaged magnetic dipole moments and magnetic size polydispersities were determined from the curves according to Chantrell et al. (1978). Saturation magnetization values were calculated by dividing the average dipole moment by the average particle volume from transmission electron microscopy.

### Transmission electron microscopy (TEM) and energy dispersive X-ray spectroscopy (EDX)

Carbon-coated Formvar Cu-grids (Agar Scientific) were dipped in nanoparticle dispersions and imaged on a Tecnai 12 (FEI) operating at 120 kV, equipped with a SIS CCD camera Megaview II. ITEM software (Olympus) was used to measure size distributions based on at least 200 particles. A Tecnai 20 (FEI) microscope operated at 200 kV, equipped with a field emission gun, Gatan 694 camera, and EDAX spectrometer was used for EDX analysis. For this purpose, raw nanoparticle dispersions were submitted to three washing cycles using 2-propanol to destabilize and cyclohexane to redisperse the particles. At least five different micron-sized spots and up to 20 individual nanoparticles were analyzed in each batch to determine particle composition and to test its uniformity over the batch.

### Ultraviolet–visible (UV/Vis) spectroscopy

**Fig. 1:**
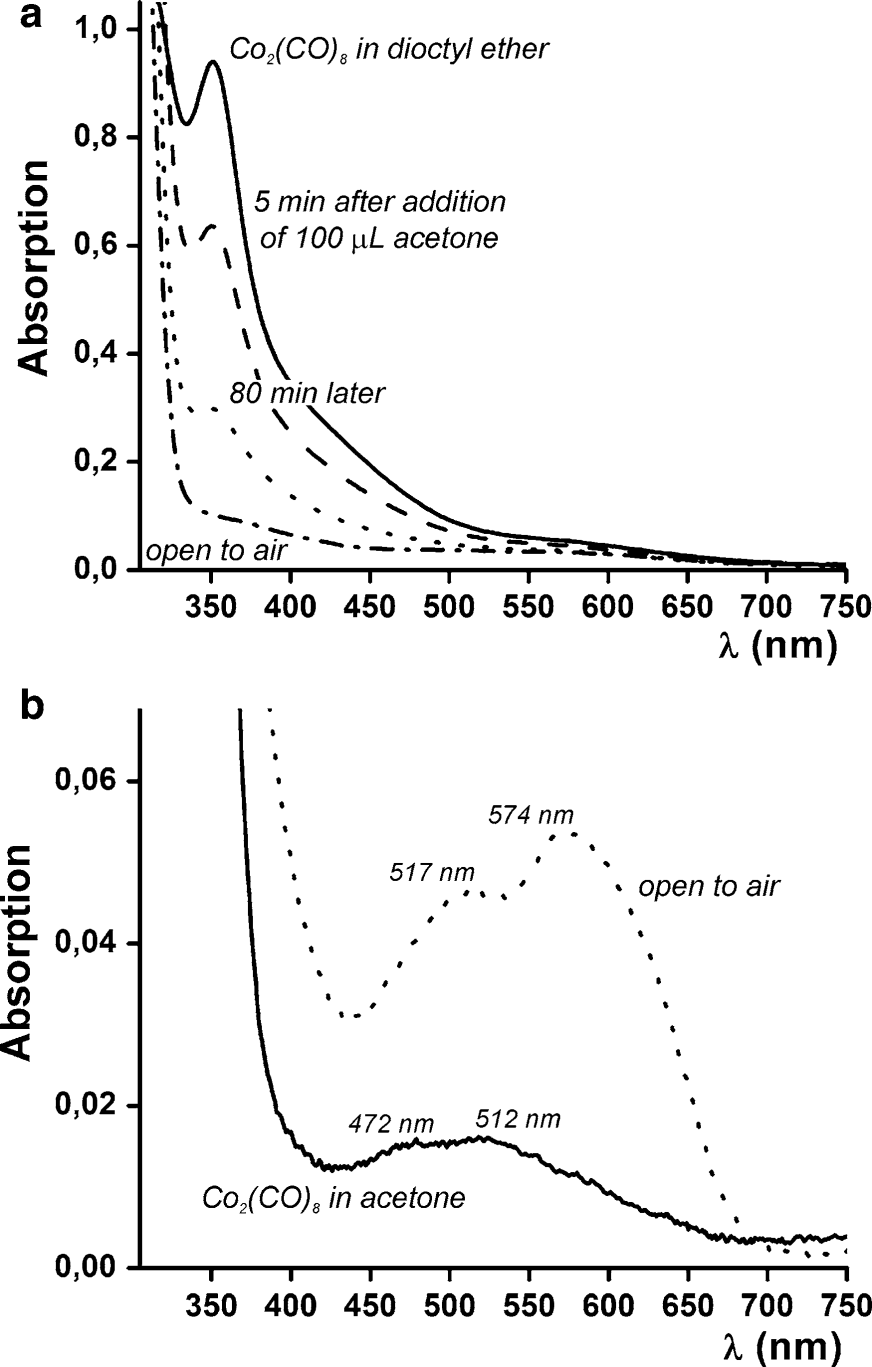
UV/Vis absorption spectra of Co_2_(CO)_8_ in **a** dioctyl ether, before and after addition of 100-μL dry acetone, resulting in Co–Co bond breaking, and **b** in anhydrous acetone, before and after exposure to air

The UV/Vis spectra in Fig. 1 and in Fig. S3c (in Online Resource 1) were acquired on a Perkin-Elmer 950 spectrometer, making use of quartz airtight cuvettes. Samples were loaded in the glove box. A Varian Cary 50 Conc spectrometer was used to acquire the remaining spectra in Online Resource 1. Samples measured on this machine were exposed to air while measuring. UV/Vis spectra of the raw syntheses can indicate the presence of *3d* transition metal containing molecular species. They display (weak) absorption features in the UV/Vis regime because of discrete *d*–*d* transitions. In contrast, nanoparticles containing hundreds to thousands of atoms are expected to form continuous *d*-bands like the bulk systems (Lau et al. 2008) that are typically 5-eV broad (Khanna et al. 1979). When the 3*d*-bands are partly filled, as in the case of Fe, Co, and Ni, this allows a myriad of optical transitions and continuous absorption in the UV/Vis regime (Johnson and Christy 1974). Hence, continuous absorption, without the presence of specific absorption peaks, is expected throughout the whole UV/Vis regime.

### X-ray powder diffraction (XRD)

XRD diffraction patterns were acquired on a Bruker D8 Advance and a Bruker D2 Phaser diffractometer. Cobalt Kα_1,2_ X-ray tubes (λ = 1.790 Å) operating at 30 kV were used, with currents of 45 and 10 mA, respectively. Typically, data points were acquired between 40° < 2θ < 100° every 0.2° with 13 s step^−1^. XRD samples were prepared inside a glove box and enclosed in an airtight and X-ray transparent box to probe the non-oxidized as prepared metal nanoparticles.

## Results

First, UV/Vis spectroscopy will be used to demonstrate that Co_2_(CO)_8_ reacts with acetone. Next, it will be shown that the acetonation step has a strong effect on the cobalt alloy nanoparticle preparation. The tunability of Co_*x*_Ni_1−*x*_ and Co_*x*_Fe_1−*x*_ particle composition will then be addressed, before revealing the particle magnetic properties. Finally, it is shown how the crystal structure of the nanoparticles is affected by the choice and concentration of the organic ligand molecules present during synthesis. The results will be further interpreted in more general terms in the "Discussion" section.

### Acetonation of cobalt carbonyl

Our alloy nanoparticle synthesis approach relies on the pre-treatment of Co_2_(CO)_8_ with dry acetone before it is thermally decomposed. In experiments using an analytical balance, mass loss was recorded on Co_2_(CO)_8_ dissolution in acetone, corresponding to 3.1 CO molecules per Co_2_(CO)_8_. The UV/Vis spectrum of Co_2_(CO)_8_ in dioctyl ether is shown in Fig. 1a before and after addition of 100 μL of dry acetone. The initial spectrum is identical to that of Co_2_(CO)_8_ in 2-methylpentane (Abrahamson et al. 1977); the peak at 350 nm is assigned to σ → σ* transitions of Co–Co derived molecular orbitals. On addition of 100 μL of dry acetone to the 2.5-mL dioctyl ether solution, a rapid decrease of the 350-nm peak intensity occurs, indicating that Co–Co bonds are broken. Figure 1b zooms in on the part of the spectrum >350 nm, for Co_2_(CO)_8_ dissolved directly in dry acetone. A stable species exhibiting two absorption features at 472 and 517 nm is observed, which is assigned to ^4^T_1g_ → ^4^T_1g_(P) transitions in high-spin octahedrally coordinated Co^2+^ 3*d* ^7^ species (Bayliss and McRae 1954; Lever 1984). This is the species from which we start the nanoparticle alloy synthesis. It is different from the species formed when the solution is exposed to air or oxygen, which would exhibit features at 512 and 574 nm because of charge transfer transitions due to O_2_ adsorption on the octahedrally coordinated Co^2+^ cations (Semenov et al. 2002). No relevant solvent effects were observed for any of the other metal precursors used in this study (see Fig. S3 in Online Resource 1).

### Beneficial effect of cobalt carbonyl acetonation on nanoparticle alloy synthesis

**Fig. 2:**
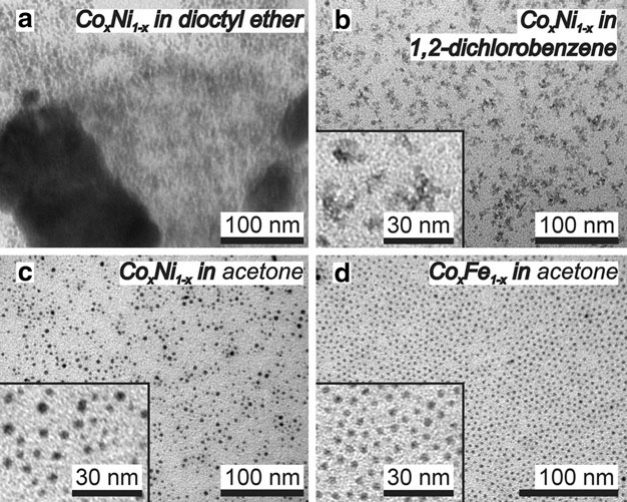
TEM pictures of the Co–Ni synthesis products when anhydrous **a** dioctyl ether, **b** dichlorobenzene, or **c** acetone were used to dissolve the Co_2_(CO)_8_ and Ni(acac)_2_ before thermal decomposition in dioctyl ether. With acetone only 4–10 nm nanoparticles were obtained. **d** The a priori acetonation of Co_2_(CO)_8_ also yields small Co–Fe nanoparticles

To dissolve the organometallic precursors prior to injection into hot dioctyl ether with OA and TOPO, the best solvent was acetone. Other precursor solvents like dioctyl ether or dichlorobenzene did not result in well-defined nanoparticles. TEM pictures of raw Co_*x*_Ni_1−*x*_ synthesis products are shown in Fig. 2. With dioctyl ether as the precursor solvent, the product consisted of polydisperse nanoparticles and irregularly shaped nickel crystals (determined by EDX) up to hundreds of nanometers in diameter (Fig. 2a). With dichlorobenzene as the precursor solvent, nanoflakes were obtained (Fig. 2b), and a UV/Vis transition was found around 670 nm, indicating the presence of residual molecular transition metal species (Fig. S4 in Online Resource 1). With acetone as the precursor solvent, spherical Co_*x*_Ni_1−*x*_ (Fig. 2c) and Co_*x*_Fe_1−*x*_ (Fig. 2d) nanoparticles were obtained and no organometallic remnants of the precursors were detected by UV/Vis; the success of acetone is ascribed to the disproportionation of Co_2_(CO)_8_, presented in the previous section.

### Tunability of nanoparticle alloy composition

**Fig. 3:**
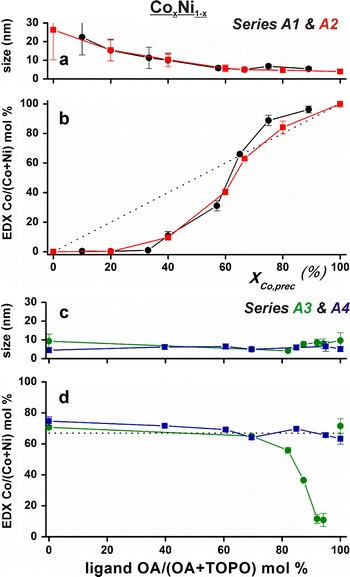
**a** TEM Co_*x*_Ni_1−*x*_ particle sizes and **b** EDX compositions versus the precursor Co/(Co + Ni) ratio in series A1 (*black circles*) and A2 (*red* *squares*). **c**, **d** Show similar curves for series A3 (*green circles*) and A4 (*blue squares*). Through series A3, the accessible particle composition could be extended to all cobalt–nickel ratios. The *dotted lines* in **b** and **d** are guides-to-the-eye corresponding to total metal precursor incorporation into the nanoparticles. (Color figure online)

Tunability of Co_*x*_Ni_1−*x*_ nanoparticle alloy composition was realized across the entire range from pure cobalt to pure nickel. This was done by systematic variation of the concentrations of the organometallic precursors and organic ligands. Figure 3 presents the average sizes and compositions as determined by TEM–EDX (additional data are provided in Table S1 and Fig. S5–6 in Online Resource 1). Figure 3a quantifies how the size and polydispersity of the nickel-rich particles decreases when the Co/Ni precursor ratio was varied at constant surfactant concentrations (series A1 and A2). Figure 3b shows the metal-to-metal ratio measured with EDX versus the metal-to-metal ratio used during nanoparticle synthesis. Cobalt-rich nanoparticles (*X*_Co,prec_ > 65 %) contained more cobalt than expected from the reactant ratio, whereas nanoparticles with less cobalt contained even less than expected from the reactant ratio. Figure 3c, d shows the results for constant amounts of Co and Ni precursors and variable amounts of surfactants, all resulting in diameters from 4 to 10 nm (series A3 and A4). Figure 3d shows that for a Co/(Co + Ni) precursor ratio of 67 %, the same ratio ends up in the nanoalloys for all ligand ratios OA/(OA + TOPO) ≤ 70 %. On increasing the amount of OA further in series A3, the nanoparticles became increasingly nickel-rich, making more Co_*x*_Ni_1−*x*_ compositions accessible. The corresponding UV/Vis spectra, at ligand ratios OA/(OA + TOPO) > 70 %, showed that the formation of these nickel-rich particles occurs at the expense of the formation of molecular species, likely cobalt-oleate (see Fig. S6c in Online Resource 1).

**Fig. 4:**
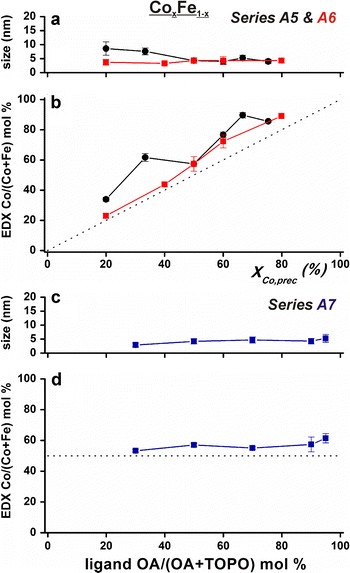
**a** TEM Co_*x*_Fe_1−*x*_ particle sizes and **b** EDX compositions versus the precursor Co/(Co + Fe) ratio in series A5 (*black circles*) and A6 (*red squares*). **c**, **d** Show similar curves for series A7 (*blue squares*). All cobalt–iron compositions for small Co_*x*_Fe_1−*x*_ particles were accessible in series A6. The *dotted lines* in **b** and **d** are guides-to-the-eye corresponding to total metal precursor incorporation into the nanoparticles. (Color figure online)

Spherical Co_*x*_Fe_1−*x*_ nanoparticles between 4 and 8.5 nm could be prepared in much the same way as the Co_*x*_Ni_1−*x*_ particles (see Fig. 4a and Fig. S7–9 in Online Resource 1). The relative amounts of cobalt and iron precursors were found in approximately the same ratio inside the nanoparticles as shown in Fig. 4b, although the particles were slightly more cobalt-rich than expected. Figure. 4c, d shows that nanoparticles prepared with a Co/(Co + Fe) precursor ratio of 50 % (series A7) varied between 4 and 6 nm and that they all had an EDX-determined Co/(Co + Fe) ratio between 50 and 60 %. Changing the OA/(OA + TOPO) ratio did thus not significantly change the metal composition. Furthermore, no sharp UV/Vis absorption features were detected for the raw Co_*x*_Fe_1−*x*_ syntheses, indicating almost complete precursor incorporation in the nanoparticles.

### Magnetization of the particles

**Fig. 5:**
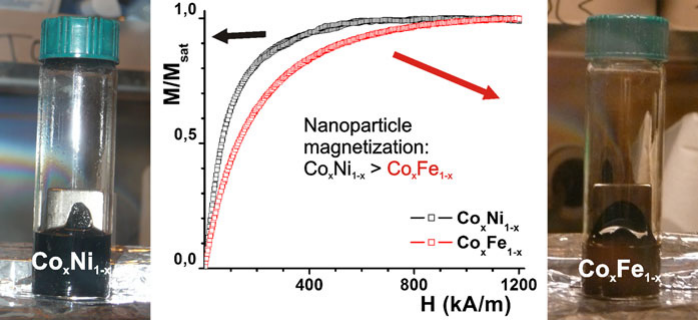
Pictures of Co_*x*_Ni_1−*x*_ (*left*) and Co_*x*_Fe_1−*x*_ (*right*) dispersions in dioctyl ether when held against a 1.3 T magnet. Typical, normalized magnetization curves of Co_*x*_Ni_1−*x*_ (*black*, *x* = 0.66) and Co_*x*_Fe_1−*x*_ (*red*, *x* = 0.53) dispersions reveal a higher magnetic dipole moment for the Co_*x*_Ni_1−*x*_ nanoparticles. (Color figure online)

The Co_*x*_Ni_1−*x*_ and Co_*x*_Fe_1−*x*_ nanoparticle dispersions behave as magnetic fluids, as shown in Fig. 5. The magnetic properties allow for size selective precipitation, and monodisperse Co_*x*_Ni_1−*x*_ and Co_*x*_Fe_1−*x*_ batches can be obtained as such (see, for example, Table S3 in Online Resource 1). Magnetization curves of multiple nanoparticle dispersions, containing ca. 1 v/v% particles in dioctyl ether, were acquired using an alternating gradient magnetometer. The average magnetic dipole moments, polydispersity in the magnetic diameter, and saturation magnetization values of 4 raw syntheses are listed in Table 1.

**Table 1:** Magnetic properties of Co_*x*_Ni_1−*x*_ and Co_*x*_Fe_1−*x*_ alloy nanoparticles

Batch	EDX Co (%)	TEM diameter (nm) polydispersity (%)	Average dipole moment (10^−20^ A m^2^)	Polydispersity of magnetic diameter (%)	Nanoparticle magnetization (kA m^−1^)
CoNi	63	5.0 (16)	4.6	38	700
CoNi	83	4.7 (27)	4.9	33	900
CoFe	55	3.7 (27)	2.1	30	380
CoFe	90	5.2 (20)	2.0	34	310

### Nanocrystalline structural phase analyses

**Fig. 6:**
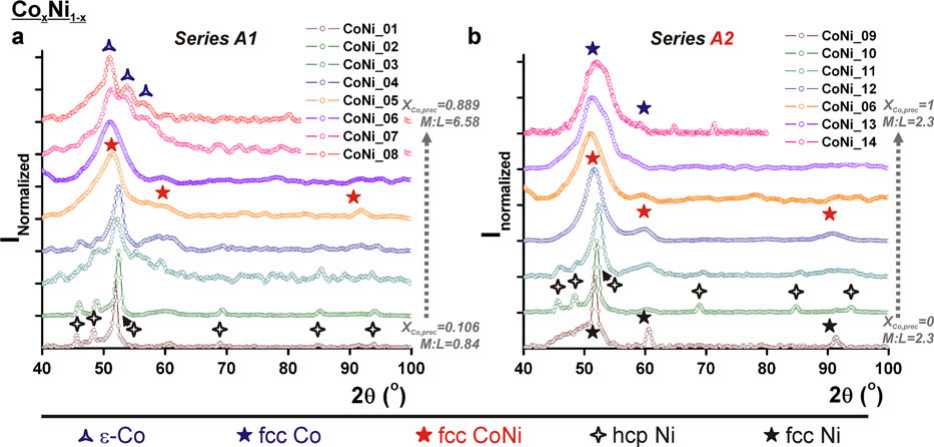
Powder X-ray diffractograms of raw Co_*x*_Ni_1−*x*_ nanoparticles, acquired in a nitrogen gas atmosphere, as made in synthesis series **a** A1 and **b** A2. The crystal phase assignments are based on the combination with the EDX results

Figure 6 shows powder X-ray diffractograms of the raw Co_*x*_Ni_1−*x*_ synthesis products of series A1 and A2 acquired in an inert environment to prevent the nanoparticles from oxidizing. Note that the phases are assigned in combination with TEM–EDX-determined compositions on individual nanoparticles as described in the "Experimental section". These analyses showed that the elemental composition from one particle to another was uniform within ten atomic percent. In Fig. 6a, a transition in series A1 was observed from nickel hexagonal close-packed (hcp) patterns for almost pure Ni aggregates, to ε-Co patterns (Murray et al. 2001) for the Co_0.96_Ni_0.04_ nanoparticles. The transitions in the XRD patterns indicate a gradually changing average nanoparticle crystal structure and since no sharp peaks were observed, the presence of larger crystals next to the nanoparticles can be excluded. In combination with the EDX results, the Co_*x*_Ni_1−*x*_ particles showing fcc peaks are assigned to a fcc CoNi phase.

Figure 6b displays a gradual change in nanoparticle crystal structure for series A2, going from fcc Ni, through hcp Ni and fcc CoNi to fcc Co. Pure nickel aggregated nanomaterials exhibited an fcc crystal structure, in contrast to almost Ni pure aggregates in series A1. For the pure cobalt nanoparticles, fcc Co was now observed in contrast to the less dense ε-Co structure for the Co_0.96_Ni_0.04_ nanoparticles in series A1. For both synthesis series A3 and A4, mainly fcc CoNi diffractograms were observed as shown in Fig. S7 (in Online Resource 1). Adding more OA (series A3), or changing the relative OA:TOPO ratios with fixed OA + TOPO amounts (series A4), did not result in crystal structure changes. Figure S9 (in Online Resource 1) shows the XRD patterns of the Co_*x*_Fe_1−*x*_ nanoparticles. All materials exhibit a fcc crystal structure, but the noise in the patterns reveals that the particles were amorphous, while the crystallinity increased for Co/(Co + Fe) ratios ≥ 90 %. The Co_*x*_Fe_1−*x*_ nanoparticle phase behavior was found to be much less complex, and less dependent on the ligands used, than that for the Co_*x*_Ni_1−*x*_ nanoparticles.

## Discussion

In the following, the presented results are systematically dealt with before ending with two general discussions. The requirements for preparing transition bimetal particles are examined in terms of the strengths of the interactions between metal atoms and organic ligands. Finally, it is addressed why these nanoparticles are suitable model systems in the search for non-noble metal based catalysts.

### Acetonation step

Hieber and Sedlmeier (1954) showed that dicobalt octacarbonyl can undergo a disproportionation reaction with Lewis bases. Typically, pyridine is found to be a base for the disproportionation of metal-carbonyls through the coordination of the nitrogen lone pair electrons. Spectrochemical series show that acetone acts as an intermediately strong Lewis base (Hieber and Sedlmeier 1954; Richmond et al. 1984). In analogy of mechanistic disproportionation studies (Hieber and Sedlmeier 1954; Sisak and Markó 1987), we propose that dicobalt octacarbonyl undergoes the following disproportionation reaction in acetone:

$$ 3Co_{2} \left( {CO} \right)_{8} + 12\left( {CH_{3} } \right)_{2} CO \to 2\left[ {Co^{2 + } \left( {\left( {CH_{3} } \right)_{2} CO} \right)_{6} } \right]\left[ {Co^{ - } \left( {CO} \right)_{4} } \right]_{2} + 8CO $$

On Co_2_(CO)_8_ dissolution in acetone, a mass loss occurred that corresponds to 3.1 CO molecules per Co_2_(CO)_8_. This is in fair agreement with the expected value of 2.7 on the basis of the proposed reaction equation. It is also supported by UV/Vis spectroscopy as shown in Fig. 1. After Co_2_(CO)_8_ acetonation, absorption due to octahedrally coordinated Co^2+^ was observed, whereas the tetracarbonyl cobaltate anions are thought to be tetrahedrally coordinated (Bühl et al. 2006) and non-absorbing in the UV/Vis regime (Semenov et al. 2002).

### Scheme to synthesize cobalt alloy nanoparticles

**Scheme 1:**
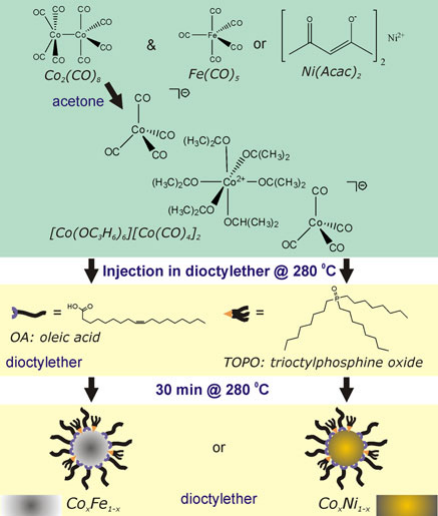
Synthesis scheme of the Co_*x*_Ni_1−*x*_ and Co_*x*_Fe_1−*x*_ alloy nanoparticles

The cobalt carbonyl acetonation product reacted with Fe(CO)_5_ and nickel(II) acetylacetonate to 4–10 nm spherical Co_*x*_Ni_1−*x*_ and Co_*x*_Fe_1−*x*_ nanoparticles with a high yield, while the use of intact Co_2_(CO)_8_ did not. This was shown in Fig. 2. On this basis, successful preparation of Co_*x*_Ni_1−*x*_ and Co_*x*_Fe_1−*x*_ nanoparticles is proposed to occur according to Scheme 1.

Although we observe that synthesis using the proposed [Co^2+^((CH_3_)_2_CO)_6_][Co^−^(CO)_4_]_2_ complex results in better alloy nanoparticles than when Co_2_(CO)_8_ is used, it remains to be revealed what the origin of this effect is. Molecular mechanistic studies of the formation of monometallic Co nanoparticles (Lagunas et al. 2006; Samia et al. 2006; de Silva et al. 2007) showed that Co_2_(CO)_8_ decomposition leads rapidly to larger Co_4_(CO)_12_ clusters and ligand-substituted analogs. On the basis of UV/Vis, we concluded that the Co–Co bonds are broken because of the acetone, which likely prevents the instantaneous formation of Co_4_(CO)_12_ intermediates. We propose that the lack of larger cobalt clusters facilitates the mixing of Co and Ni or Co and Fe atoms in alloy nanoparticles. Also, the presence of an overall neutral complex of ligated cations and carbonylate anions might favor the stabilization of mono-cobalt building blocks, for example, by a facilitated de-protonation of the oleic acid molecules in solution to form bonding oleate complexes. Such intermediates would have slower and similar reaction rates as the Fe(CO)_5_ or Ni(acac)_2_ precursors.

The failure to obtain uniform well-mixed Fe_*x*_Ni_1−*x*_ nanoparticles underpins the importance of the proposed disproportionated cobalt complex in the synthesis. The studies of Hieber and others has shown that base-induced disproportionation reactions exist for vanadium (Richmond et al. 1984), manganese (Hieber et al. 1961), iron (Hieber and Kahlen 1958), and nickel (Hieber et al. 1932) carbonyls and further studies might exploit this for the synthesis of other families of alloy nanoparticles.

### Structure, composition, and possibility of oxidation of the cobalt alloy nanoparticles

The TEM–EDX results for the Co_*x*_Ni_1−*x*_ and Co_*x*_Fe_1−*x*_ nanoparticles, as shown in Figs. 3 and 4, revealed that the obtained particles contain both metals and that the bulk-determined composition is the same in individual nanoparticles. The powder X-ray diffractograms in Fig. 6 featured only one crystallographic phase per synthesis. In combination with the TEM–EDX results, this indicates that small alloy nanoparticles with one (poly)crystalline phase per synthesis were obtained. Furthermore, because this is not a seeded growth synthesis, core–shell structures are not likely. To determine the atomic distribution within one bimetallic nanoparticle, more advanced characterization methods such as scanning transmission electron microscopy combined with electron energy loss spectroscopy would be needed (van Schooneveld et al. 2010; den Breejen et al. 2011). Nonetheless, series A1 and A2 hinted at important information on the surface composition of the nanoparticles. Small Co_*x*_Ni_1−*x*_ alloy particles were only obtained when increasingly more cobalt was added. This increased the total metal-to-ligand ratio. In a monometallic synthesis, particles normally grow larger when increasing the metal-to-ligand ratio. Here, the opposite behavior is observed and it can be explained by ligand-induced metal segregation (Menning and Chen 2009). For cobalt–nickel alloys, the surface would consist of nickel atoms in vacuum (Menning and Chen 2009). However, for oxygen atoms, it has been predicted that the adsorbate–metal interactions drive cobalt atoms to the particle surface (Menning and Chen 2009). It is also known that cobalt has a higher affinity for oleic acid than nickel. We suggest that the particles were large in order to shield the nickel atoms behind the relatively little amount of cobalt atoms that were forming the surface with the oxygen-containing ligand functional groups. In this view, the particles became smaller on addition of more cobalt, because more cobalt could sit at the particle interface. For bimetallic nanoparticles, these results show that next to the metal-to-ligand ratio, the metal–ligand affinity plays an important role in controlling their size and shape. In this view, single-crystal phase alloy nanoparticles were prepared where the first outer layer consisted of cobalt atoms in case of the Co_*x*_Ni_1−*x*_ nanoparticles.

Finally, it is noted that the examined particles are unlikely to be oxidized. They were prepared in a nitrogen atmosphere Schlenk line and stored in a nitrogen atmosphere glove box. The XRD and AGM measurements were done under exclusion from air. No oxidation-related peaks were observed in XRD, and the nanoparticle magnetic properties are indicative of the highly magnetic metals as compared with the somewhat less magnetic metal oxides. The inferior magnetic properties of the Co_*x*_Fe_1−*x*_ with respect to the Co_*x*_Ni_1−*x*_ particles might, however, be due to a slight degree of iron oxidation, undetectable by XRD. In case of oxidation, iron is likely oxidized first in Co_*x*_Fe_1−*x*_, while the oxidation of cobalt is expected to occur first in Co_*x*_Ni_1−*x*_ nanoparticles as a result of their respective oxidation potentials (Haynes and Lide 2012).

### Magnetic properties of the alloy nanoparticles

The saturation magnetization values for pure Fe, Co, and Ni solids at room temperature are 1,711, 1,424, and 485 kA m^−1^, respectively (Chikazumi 1964). In bulk alloys of iron, cobalt, and nickel, saturation magnetizations are found to be intermediate between their components (Crangle and Hallam 1963). For the bimetallic nanoparticles prepared here, the saturation magnetization values were of the same order of magnitude, indicating a good magnetic quality. They are at least as magnetic as magnetite or maghemite nanoparticles of the same size, because the latter are usually less magnetic than expected from the magnetization of about 450 kA m^−1^ for bulk magnetite or maghemite (Chikazumi 1964). The saturation magnetization values and average magnetic dipole moments of the Co_*x*_Fe_1−*x*_ particles were, however, lower than those of the Co_*x*_Ni_1−*x*_ particles. Possibly, a minor degree of iron oxidation resulted in loss of magnetization. Alternatively, it could be due to the lower degree of Co_*x*_Fe_1−*x*_ crystallinity, since crystalline defects are known to have a detrimental effect on the magnetic properties of nanoparticles (Luigjes et al. 2011). Overall, it is important to note that the effective magnetic properties for alloy nanoparticles cannot be solely predicted on the basis of the bulk magnetic properties, but effects such as crystallinity and the ease and degree of metal oxidation should be taken into account. Here, an example is reported where Co_*x*_Ni_1−*x*_ particles display superior magnetic properties to their Co_*x*_Fe_1−*x*_ analogs, while the opposite is expected based on bulk properties alone.

### Ligand tunability of the crystal structure

**Fig. 7:**
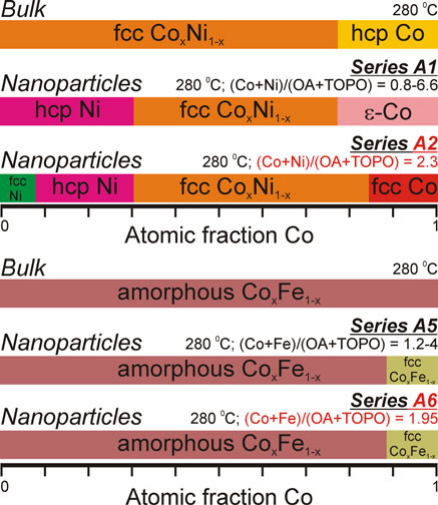
Summary of the phase behavior of Co_*x*_Ni_1−*x*_ and Co_*x*_Fe_1−*x*_ nanoparticles when synthesized at 280 °C following the described wet-chemical synthesis route. The phase behavior of bulk metal alloys (Baker 1992) is shown for comparison

Figure 7 summarizes the phase behavior of the Co_*x*_Ni_1−*x*_ and Co_*x*_Fe_1−*x*_ alloys obtained at the synthesis temperature of 280 °C and indicates the stable phases of the bulk alloys for comparison (Baker 1992). The figure combines the determined crystalline phases in the nanoparticles (based on EDX and XRD as shown in Fig. 6) with the respective synthesis parameters. It must be noted that Fig. 7 is based on the here presented 40 syntheses and that the exact elemental fractions at which the transitions occur, or the discreteness with which the boundaries are drawn, are subject to an error of a few percent because of the limited amount of data points. However, while the crystalline phase behavior of bulk alloys is typically described as a function of temperature only, we summarized in Fig. 7 that the phase behavior of the Co_*x*_Ni_1−*x*_ and Co_*x*_Fe_1−*x*_ nanocrystalline alloys is a function of the type of ligands used, their mutual ratios, and their concentration ratios with respect to the metals. These are essential state variables, and additional ones compared with bulk systems, when describing the alloy nanoparticle phase behavior. It is striking, for example, that both ε-Co and fcc Co nanoparticles, having a less dense respectively a denser structure than hcp Co, can be obtained in the Co_*x*_Ni_1−*x*_ synthesis series by varying the reactant concentrations. Figure 7 shows the first detailed description of the Co_*x*_Ni_1−*x*_ and Co_*x*_Fe_1−*x*_ phase behavior as a function of oleic acid and trioctylphosphine oxide ligands. To fully control bimetallic particle formation at the nanometer scale in general, it is recommended to study their syntheses in the systematic way as shown here.

### General energetic considerations of preparing alloy nanoparticles

The previous discussion raises the question what the general requirements are with respect to the strength of the metal–metal, ligand–ligand, and metal–ligand interactions. In designing an alloy nanoparticle synthesis method, one can first consider metal–metal interactions. The bulk alloy phase diagrams indicated that Co/Ni and Co/Fe are miscible over a large range of elemental ratios at the synthesis temperature of 280 °C (Baker 1992). Furthermore, the enthalpies of formation were favorable. For Co_*x*_Fe_1−*x*_ bulk systems, experimental and calculated enthalpies of formation for ordered or interstitial alloys were reported to be respectively −10 to −1 kJ mol^−1^ and −22 to −1 kJ mol^−1^ for all *x* (de Boer et al. 1989). For Co_*x*_Ni_1−*x*_ bulk systems, these were found to be respectively 0 and −13 to +3 kJ mol^−1^ for all *x* (de Boer et al. 1989).

Secondly, the metal–ligand interactions should be considered. In the synthesis of nanoparticles, extensive use is made of a few ligands that include phosphines (R_3_P), phosphites (R_3_PO), acids (RCOOH), alcohols (ROH), amines (RNH_2_), and thiols (RSH) (Donega 2011). An assumption in this study was that particle stability would be favored if the average dissociation energies for M_*x*_–M_*y*_ ~ M_*x*_–M_*x*_ ~ M_*y*_–M_*y*_ ≥ M_*x*/*y*_–L_a_, and M_*x*/*y*_–L_b_. Although solvation and surface energy arguments are neglected here, the idea of the requirement is that metal atoms would be prone to leaching from the nanoparticle surface by strongly binding ligands. Literature values of dissociation energies of cationic species Fe^+^–Fe and Co^+^–Fe are ca. 260 kJ mol^−1^ (Haynes and Lide 2012), between M^+^–S with M = Fe, Co, Ni they are 250–260 kJ mol^−1^ (Marks 1990), and between M^+^–NH_2_ they are 232–235 kJ mol^−1^ for Ni, 247–260 kJ mol^−1^ for Co and 280 kJ mol^−1^ for Fe (Marks 1990; Haynes and Lide 2012). Based on this, it was decided not to use thiols or amines in the synthesis of Co_*x*_Ni_1−*x*_ and Co_*x*_Fe_1−*x*_ nanoparticles. Although amines are usually applied in nickel nanoparticle synthesis, we verified by ligand-exchange tests on pure ε-Co nanoparticles, prepared by the Puntes method (Puntes et al. 2001), that these aggregated and even dissolved on post-synthesis addition of dodecylamine and 1-dodecanethiol, respectively (see Fig. S2 in Online Resource 1). Kitaev (2008) also noted the (partial) dissolution of cobalt nanoparticles on thiol addition. Instead, OA and TOPO molecules that both act as *soft* Lewis bases on the *hard* Lewis acid transition metals were chosen for their mild binding energies with cobalt and iron.

The third consideration concerns the dilemma between bond strength and amount of ligands used in the bimetal nanoparticle synthesis. Ligands, here OA and TOPO, which are just right to form cobalt and iron nanoparticles cannot prevent nickel from aggregating when used in equally low concentrations. On the other hand, ligands, such as the amines that bind strongly with nickel, dissolve the cobalt and iron into molecular complexes. The preparation of composition tunable transition metal nanoparticles, from metals with seemingly incompatible ligand affinity, can be realized by the use of one of the ligands at higher concentrations, at the expense of product yield. For example, the low binding strength of OA and TOPO initially prevented the formation of Co_*x*_Ni_1−*x*_ particles with low cobalt content in series A1 and A2, but by adding more ligands, such particles were obtained in series A3, albeit together with cobalt molecular complexes and thus incomplete conversion.

### Model systems for non-noble metal based catalysis

Solution prepared nanoparticles are capped with ligands to prevent them from aggregation. These ligands might seriously lower their activity in a catalytic reaction that occurs at the particle surface. In this respect, it is more useful to prepare nanoparticles on a support material through classic preparation routes used in heterogeneous catalysis. The advantage of using colloidal nanoparticles is, however, that the size and composition of all particles is readily controlled, as, for example, shown in this study. These particles, when coated with different ligands and consisting of different metal atoms, are then suitable model systems to study the interactions of alloys with the chemical intermediates of catalyzed reactions. Especially, the Newns–Anderson model predicts the adsorbate bond dissociation energies and adsorbate-induced metal segregation in bimetallic systems, as a function of the metal *d*-band center, providing a predictive framework for active non-noble metal catalysts (Nilsson et al. 2008; Menning and Chen 2009; Nørskov et al. 2011).

## Conclusions

A generally applicable organometallic synthesis route, based on the reaction of Co_2_(CO)_8_ with acetone, is reported for the synthesis of 4–10 nm Co_*x*_Ni_1−*x*_ and Co_*x*_Fe_1−*x*_ nanoparticles with tunable elemental compositions. Based on the results of seven series of syntheses where the metal precursor concentrations and ligand type and concentrations were varied, insights intrinsic to the size, composition, and phase behavior of stable bimetallic alloy nanoparticles has been obtained. These basic insights will provide guidelines for the wet-chemical synthesis of yet unmade bimetallic alloy nanoparticles. We further envisage that the well-defined Co_*x*_Ni_1−*x*_ and Co_*x*_Fe_1−*x*_ nanoparticles are suitable prototypes to test the Newns–Anderson model as used in catalysis.

## Electronic supplementary material

Below is the link to the electronic supplementary material.

Supplementary Material 1 (1490 kb)
