# Supplementary material for: Composition tunable cobalt–nickel and cobalt–iron alloy nanoparticles below 10 nm synthesized using acetonated cobalt carbonyl
Source: J Nanopart Res. 2012 Jul 4;14(8):991. doi: 10.1007/s11051-012-0991-5 (PMC3422617; doi:10.1007/s11051-012-0991-5)
Supplement: Supplementary file 1 — Supplementary Material 1 (1490 kb) [file 11051_2012_991_MOESM1_ESM.pdf]

Electronic Supplementary Material

Composition tunable cobalt-nickel and cobalt-iron alloy nanoparticles below 10 nanometer synthesized using acetonated cobalt carbonyl

Matti M. van Schooneveld, Carlos Campos-Cuerva, Jeroen Pet, Johannes D. Meeldijk, Jos van Rijssel, Andries Meijerink, Ben H. Erné, and Frank M.F. de Groot

Matti M. van Schooneveld, Carlos Campos-Cuerva, Jeroen Pet, Johannes D. Meeldijk, Frank M.F. de Groot
Inorganic Chemistry & Catalysis, Debye Institute for Nanomaterials Science, Utrecht University, Universiteitsweg 99, 3584 CG, The Netherlands.

Jos van Rijssel, Ben H. Erné
Van 't Hoff Laboratory for Physical & Colloid Chemistry, Debye Institute for Nanomaterials Science, Utrecht University, Padualaan 8, 3584 CH Utrecht, The Netherlands.

Andries Meijerink
Condensed Matter & Interfaces, Debye Institute for Nanomaterials Science, Utrecht University, P.O. Box 80000, 3508 TA Utrecht, The Netherlands.

Corresponding authors: Matti M. van Schooneveld and Frank M.F. de Groot
e-mail: M.M.vanSchooneveld@gmail.com; F.M.F.deGroot@uu.nl
Phone: 00 31 30 253 7400; Fax: 00 31 30 251 1027.

List of contents

*Adapted Synthesis Flask & **Fig. S1*** S2
*Ligand-Exchange on 5 nm pure Co Nanoparticles & **Fig. S2*** S2
***Table S1.** Co_xNi_{1-x} nanoparticle synthesis and characterization* S3
***Table S2.** Co_xFe_{1-x} nanoparticle synthesis and characterization* S4
***Table S3.** Size selective precipitation of magnetic Co_xNi_{1-x} and Co_xFe_{1-x} nanoparticles* S4
*Precursor and Solvent Effects on the Synthesis of Co_xNi_{1-x} and Co_xFe_{1-x} Particles & **Fig. S3-4*** S5
*TEM of Co_xNi_{1-x} Nanoparticles Synthesized in Series A2 & **Fig. S5*** S6
*UV/Vis and XRD of the Co_xNi_{1-x} Nanoparticles Synthesized in Series A1-A4 & **Fig. S6*** S7
*Attempted Synthesis of Fe_xNi_{1-x} Nanoparticles & **Fig. S7*** S8
*TEM of Co_xFe_{1-x} Nanoparticles Made in Series A6 & **Fig. S8*** S8
*UV/Vis and XRD of the Co_xFe_{1-x} Nanoparticles Synthesized in Series A5-A7& **Fig. S9*** S9

Adapted Synthesis Flask

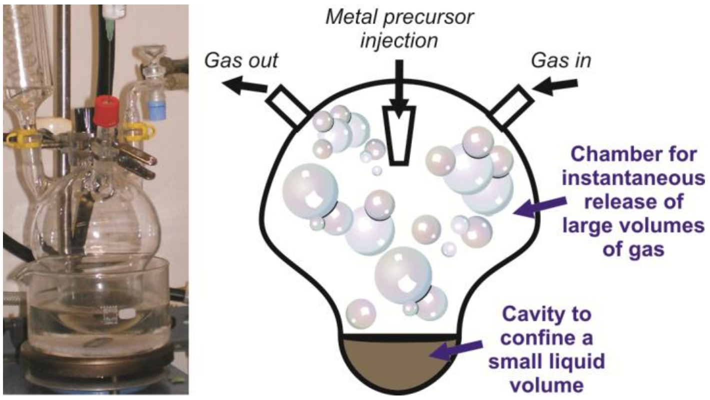

Fig. S1. Picture (left) and schematic representation (right) of the homemade Schlenk vessel, designed for reactions in a small liquid volume with substantial and instantaneous gas release. The flask consists of a spherical bulge with a small diameter at the bottom of the flask and a large volume above it. This design allows the sudden release of CO gas upon precursor injection to expand within the synthesis flask only. With a theoretical maximum gas release of 800 mL for an ideal gas at 280 °C for the here presented syntheses, the relatively small amount of liquid (~12 mL) is then kept in the bulge of the flask as a true liquid and not spread as a film.

Ligand-Exchange on 5 nm pure Co Nanoparticles

1-dodecanethiol (≥98%), dodecylamine (99%), and 1-dodecanol (98%) were purchased from Aldrich. To three 1.5 mL batches of a 0.5 nmol/mL dispersion of 5.6 ± 0.7 nm and pure ε-Co nanoparticles in 1,2-dichlorobenzene, 0.18, 0.36, and 0.72 mmol 1-dodecanol was added respectively. The same was repeated with dodecylamine and 1-dodecanethiol instead of 1-dodecanol. After two days the samples were characterized with TEM and UV/Vis absorption spectroscopy to examine the effect of the ligand exchange.

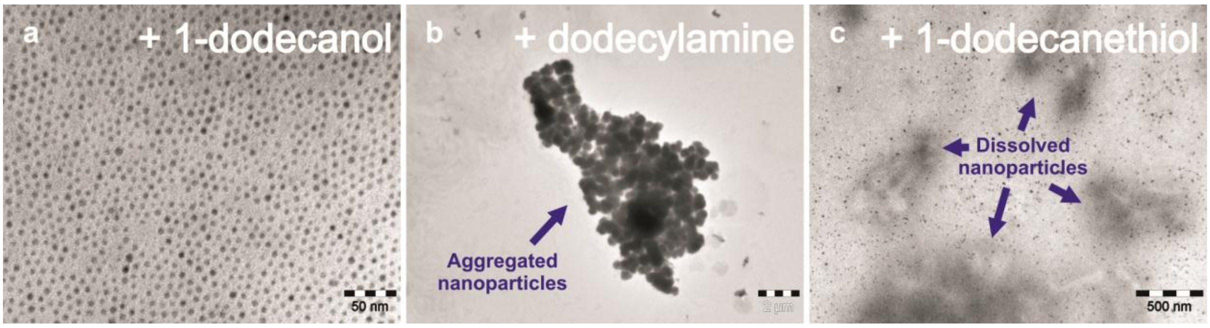

Fig. S2. TEM pictures of 5.6 ± 0.7 nm ε-Co nanoparticles in 1,2-dichlorobenzene to which 0.36 mmol (a) 1-dodecanol, (b) 1-dodecanamine, and (c) 1-dodecanethiol were added. While the alcohol stabilized the Co nanoparticles, the amines led to partial nanoparticle aggregation into micron sized structures, and the thiols partially dissolved the nanoparticles. In UV/Vis absorption spectra (not shown) three absorption peaks were observed for the thiol-exchanged batch at ~315, 402, and 509 nm confirming the presence of molecular Co species in solution

Table S1. Co_xNi_{1-x} nanoparticle synthesis and characterization. Amounts of reactants and their molar ratios in the different Co_xNi_{1-x} nanoparticle syntheses, as well as the TEM average size, polydispersity and EDX mean composition of the obtained nanoparticles.

**Batch**	**$Co_2(CO)_8$ (g)**	**$Ni(acac)_2$ (g)**	**mol % Co/(Co+Ni)**	**OA (g)**	**TOPO (g)**	**mol % OA/(OA +TOPO)**	**M:L[a] molar ratio**	**TEM <D>±S.D.[b] (nm)**	**Poly-dispersity[c] (%)**	**EDX Co/(Co+Ni) ± S.D.[a] (%)**
**Series A1: mol Ni, TOPO, OA constant; mol Co variable**										
CoNi_01	0.023(4)	0.297(5)	10.6	0.305(8)	0.179(3)	70.0	0.84	22.4 ± 9.6	43	0 ± 2
CoNi_02	0.049(9)	0.306(8)	19.6	0.298(7)	0.186(5)	68.7	0.97	15.3 ± 5.6	37	0 ± 2
CoNi_03	0.100(0)	0.300(0)	33.4	0.320(0)	0.180(0)	70.9	1.10	11.3 ± 5.7	50	1 ± 1
CoNi_04	0.134(1)	0.299(6)	40.2	0.299(2)	0.181(5)	69.3	1.28	10.0 ± 3.4	34	11 ± 3
CoNi_05	0.270(0)	0.300(0)	57.5	0.300(0)	0.180(0)	69.5	1.80	5.8 ± 1.1	19	31 ± 3
CoNi_06	0.400(0)	0.300(0)	66.7	0.30(0)	0.18(0)	69.5	2.30	5.0 ± 0.8	16	64 ± 3
CoNi_07	0.600(0)	0.310(0)	74.4	0.300(0)	0.180(0)	69.5	3.09	6.8 ± 1.2	18	89 ± 4
CoNi_08	1.600(0)	0.300(0)	88.9	0.320(0)	0.180(0)	70.9	6.58	5.4 ± 1.0	18	96 ± 2
**Series A2: mol Co+Ni, TOPO, OA constant; Co/Ni ratio variable**										
CoNi_09	0	0.919(4)	0	0.299(4)	0.180(3)	69.4	2.34	26.3 ± 16.1	61	0 ± 2
CoNi_10	0.119(4)	0.721(5)	19.9	0.299(7)	0.183(0)	69.2	2.29	15.5 ± 5.8	37	0 ± 2
CoNi_11	0.239(7)	0.541(1)	40.0	0.301(4)	0.180(6)	69.6	2.29	10.3 ± 3.7	36	10 ± 1
CoNi_12	0.359(9)	0.361(5)	59.9	0.302(5)	0.179(8)	69.7	2.29	5.7 ± 1.8	32	41 ± 2
CoNi_06	0.40(0)	0.30(0)	66.7	0.30(0)	0.18(0)	69.5	2.30	5.0 ± 0.8	16	64 ± 3
CoNi_13	0.479(8)	0.180(6)	80.0	0.299(4)	0.180(1)	69.5	2.30	4.7 ± 1.3	27	84 ± 4
CoNi_14	0.598(9)	0	100	0.300(8)	0.180(8)	69.5	2.29	3.9 ± 1.2	30	100 ± 2
**Series A3: mol Ni, Co, TOPO constant; mol OA variable**										
CoNi_15	0.400(0)	0.320(0)	65.2	0	0.180(0)	0	7.70	9.3 ± 3.8	40	71 ± 1
CoNi_06	0.400(0)	0.300(0)	66.7	0.30(0)	0.18(0)	69.5	2.30	5.0 ± 0.8	16	64 ± 3
CoNi_16	0.408(2)	0.298(7)	67.3	0.598(5)	0.179(9)	82.0	1.37	4.2 ± 0.9	22	56 ± 2
CoNi_17	0.410(0)	0.300(0)	67.3	0.910(0)	0.180(0)	87.4	0.97	7.7 ± 1.4	18	37 ± 1
CoNi_18	0.399(0)	0.299(3)	66.7	1.513(7)	0.179(9)	92.0	0.60	8.6 ± 1.8	20	12 ± 3
CoNi_19	0.420(0)	0.300(0)	67.8	2.120(0)	0.180(0)	94.2	0.45	8.0 ± 2.5	31	11 ± 4
CoNi_20	0.400(0)	0.300(0)	66.7	0.300(0)	0	100	3.30	9.7 ± 3.2	32	72 ± 5
**Series A4: mol Ni, Co, TOPO+OA constant; OA/TOPO variable**										
CoNi_21	0.401(7)	0.299(7)	66.8	0	0.590(8)	0	2.30	4.5 ± 1.5	33	75 ± 3
CoNi_22	0.400(2)	0.300(6)	66.7	0.171(1)	0.355(2)	39.7	2.30	6.2 ± 1.4	23	72 ± 1
CoNi_23	0.400(1)	0.300(6)	66.7	0.266(0)	0.236(4)	60.6	2.26	6.5 ± 1.4	22	69 ± 1
CoNi_06	0.40(0)	0.30(0)	66.7	0.30(0)	0.18(0)	69.5	2.30	5.0 ± 0.8	16	64 ± 3
CoNi_24	0.400(4)	0.300(3)	66.7	0.365(9)	0.089(2)	84.9	2.30	5.9 ± 1.3	22	70 ± 1
CoNi_25	0.401(4)	0.301(3)	66.7	0.411(5)	0.029(8)	95.0	2.30	6.5 ± 2.6	39	66 ± 1
CoNi_26	0.398(7)	0.300(9)	66.6	0.430(7)	0	100	2.30	5.1 ± 1.4	27	63 ± 4

[a.] M:L = metal:ligand molar ratio, defined by (Co+Ni)/(TOPO+OA). The studied variables in the different series are underlined. [b.] <D> = average diameter & S.D. = standard deviation; [c.] Polydispersity is defined as S.D./<D>*100%

Table S2. Co_xFe_{1-x} nanoparticle synthesis and characterization. Amounts of reactants and their molar ratios in the different Co_xFe_{1-x} nanoparticle syntheses, as well as the TEM average size, polydispersity and EDX mean composition of the obtained nanoparticles.

Batch	$Co_2(CO)_8$ (g)	$Fe(CO)_5$ (g)	mol % Co/(Co+Fe)	OA (g)	TOPO (g)	mol % OA/(OA +TOPO)	M:L[a] molar ratio	TEM <D>±S.D.[b] (nm)	Poly-dispersity[c] (%)	EDX Co/(Co+Fe) ± S.D.[a] (%)
**Series A5: mol Fe, TOPO, OA constant; mol Co variable**										
CoFe_01	0.087(0)	0.399(0)	20.0	0.530(0)	0.080(0)	90.1	1.22	8.6 ± 2.4	28	34 ± 1
CoFe_02	0.174(8)	0.400(0)	33.4	0.540(8)	0.080(8)	90.2	1.44	7.6 ± 1.2	16	62 ± 3
CoFe_03	0.348(0)	0.399(0)	50.0	0.530(0)	0.080(0)	90.1	1.95	4.3 ± 1.0	24	57 ± 5
CoFe_04	0.521(4)	0.398(0)	60.0	0.534(0)	0.079(7)	90.2	2.42	4.0 ± 0.9	24	77 ± 2
CoFe_05	0.699(3)	0.400(8)	66.7	0.530(5)	0.080(0)	90.1	2.94	5.2 ± 1.0	20	90 ± 2
CoFe_06	1.074(8)	0.402(1)	75.4	0.535(0)	0.080(1)	90.1	3.97	4.0 ± 0.8	20	86 ± 1
**Series A6: mol Co+Fe, TOPO, OA constant; Co/Fe ratio variable**										
CoFe_07	0.138(5)	0.636(5)	20.0	0.530(6)	0.081(8)	89.9	1.94	3.7 ± 1.0	27	23 ± 2
CoFe_08	0.278(0)	0.478(0)	40.0	0.531(0)	0.079(9)	90.1	1.95	3.3 ± 0.3	10	44 ± 1
CoFe_03	0.348(0)	0.399(0)	50.0	0.530(0)	0.080(0)	90.1	1.95	4.3 ± 1.0	24	57 ± 5
CoFe_09	0.418(1)	0.319(4)	60.0	0.530(6)	0.081(3)	89.9	1.95	4.3 ± 1.4	33	72 ± 4
CoFe_10	0.558(5)	0.160(7)	80.0	0.531(3)	0.080(2)	90.1	1.96	4.3 ± 0.9	27	89 ± 1
**Series A7: mol Fe, Co, TOPO+OA constant; OA/TOPO variable**										
CoFe_11	0.348(0)	0.399(0)	50.0	0.176(5)	0.563(7)	30.0	1.96	2.9 ± 0.7	24	53 ± 1
CoFe_12	0.347(3)	0.398(6)	50.0	0.293(8)	0.402(8)	50.0	1.95	4.2 ± 0.9	21	57 ± 1
CoFe_13	0.350(0)	0.400(7)	50.0	0.412(1)	0.241(7)	70.0	1.96	4.7 ± 1.1	23	55 ± 1
CoFe_03	0.348(0)	0.399(0)	50.0	0.530(0)	0.080(0)	90.1	1.95	4.3 ± 1.0	24	57 ± 5
CoFe_14	0.347(2)	0.398(1)	50.0	0.558(7)	0.040(0)	95.0	1.95	5.3 ± 1.4	26	61 ± 3

[a.] M:L = metal:ligand molar ratio, defined by (Co+Fe)/(TOPO+OA). The studied variables in the different series are underlined. [b.] <D> = average diameter & S.D. = standard deviation; [c.] Polydispersity is defined as S.D./<D>*100%

Table S3. Size selective precipitation of magnetic Co_xNi_{1-x} and Co_xFe_{1-x} nanoparticles

Batch	TEM <D> ± S.D.[a] (nm)	
	**CoNi @ 260 °C**	**CoFe_15**
**Raw material**	6.4 ± 1.0	6.0 ± 2.0
**Fraction 1**	6.6 ± 1.1	8.1 ± 1.4
**Fraction 2**	5.6 ± 0.6	4.2 ± 0.7
**Fraction 3**	4.9 ± 0.8	3.0 ± 0.6
**Fraction 4**	-	2.1 ± 0.6

[a.] D = diameter & S.D. = standard deviation

Precursor and Solvent Effects on the Synthesis of Co_xNi_{1-x} and Co_xFe_{1-x} Particles

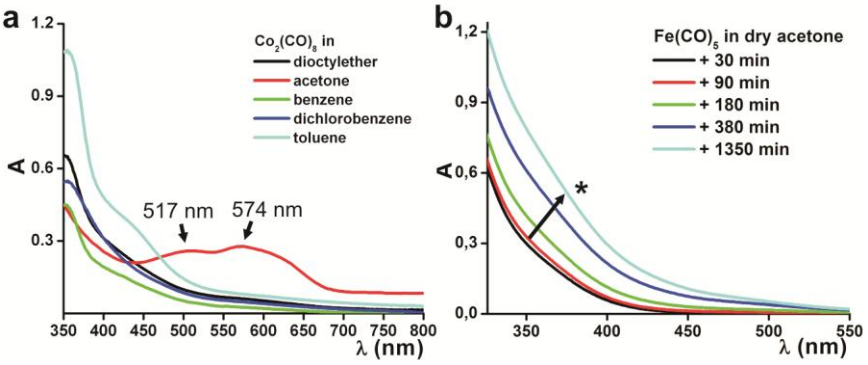

Fig. S3. Effect of different solvents on the stability of the used metal precursors as studied with UV/Vis absorption spectroscopy. Spectra of (a) $Co_2(CO)_8$ in *air-exposed* dioctyl ether, acetone, benzene, dichlorobenzene, and toluene. Spectra in the same solutions were taken for $Co(acac)_3$, $Ni(acac)_2$, and $Fe(CO)_5$ (not shown). It can be concluded from these experiments that only $Co_2(CO)_8$ undergoes a reaction with one of the solvents, being acetone. The acetone spectrum shows peaks at 517 and 574 nm from oxygenated Co^{2+} cations as discussed in the main text. In panel (b) the effect of dissolving $Fe(CO)_5$ in *anhydrous* acetone in time is shown. Within the first 90 min hardly any changes occurred. Later, the spectra showed an increased absorption throughout the full UV/Vis regime (indicated with an asterisk), suggesting the clustering of multiple iron atoms. Most importantly, it is concluded that $Fe(CO)_5$ does hardly undergo a (disproportionation) reaction with acetone in the time frame relevant (30 min) for the Co_xFe_{1-x} syntheses. The iron precursor is thus, contrary to the acetonated $Co_2(CO)_8$, added as $Fe(CO)_5$ to the OA and TOPO containing dioctyl ether

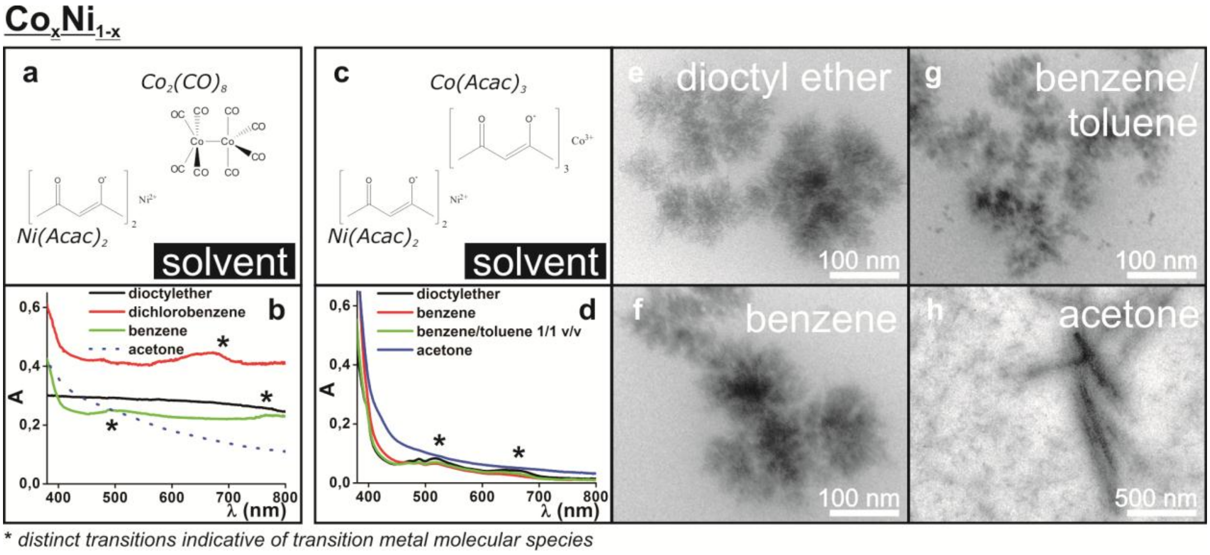

Fig. S4. Effect of different metal precursors and solvents on the products obtained in attempted Co_xNi_{1-x} nanoparticle syntheses. (a) Structure formulas of $Co_2(CO)_8$ and $Ni(acac)_2$, the precursor molecules used in the syntheses shown in Fig. 2 of the main text, and (b) the corresponding UV/Vis absorption spectra of these syntheses. Distinct UV/Vis absorption features, indicated with asterisks, are indicative of molecular transition metal species and thus incomplete precursor-to-nanoparticle conversion. (c) Structure formulas of $Co(acac)_3$ and $Ni(acac)_2$, used as alternative metal precursor molecules in a synthesis series aimed at making Co_xNi_{1-x} nanoparticles when using different precursor solvents. (d) UV/Vis spectra of the obtained synthesis dispersions. (e-h) TEM pictures of the raw syntheses products. Using acetone flake-like objects were observed on the nano scale and elongated structures on the micron scale. In none of the solvents it was possible to obtain Co_xNi_{1-x} spherical nanoparticles from $Co(acac)_3$ and $Ni(acac)_2$

Transmission Electron Micrographs of Co_xNi_{1-x} Nanoparticles Synthesized in Series A2

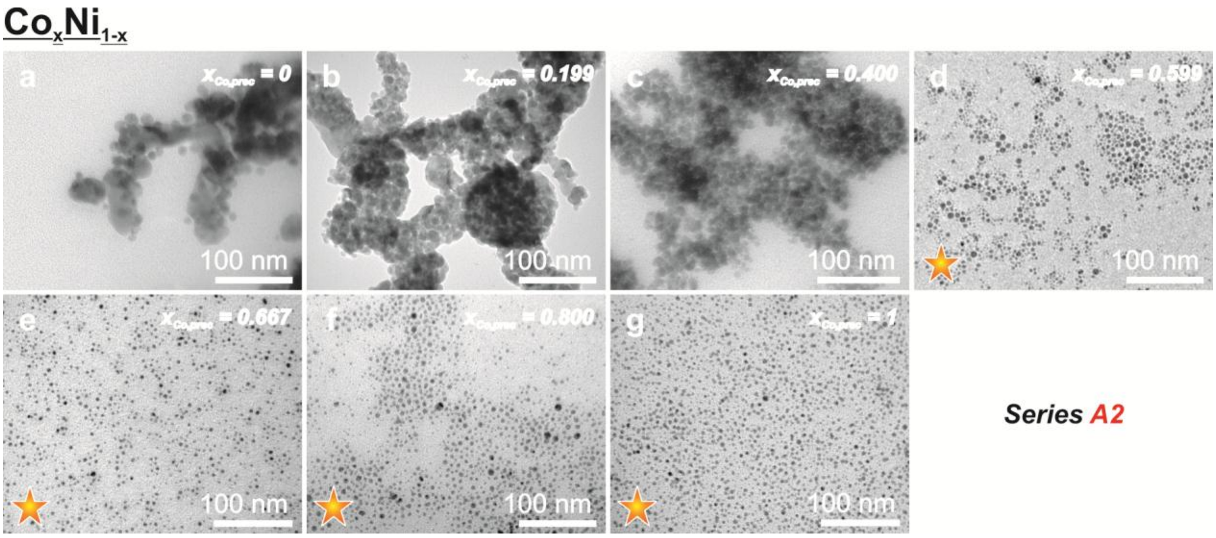

Fig. S5. TEM images of Co_xNi_{1-x} alloy nanoparticles as prepared in synthesis series A2. In series A2 a fixed OA/(OA+TOPO) and (Co+Ni)/(OA+TOPO), or M:L, ratio of 0.7 and 2.3 were used respectively, while the Co/(Co+Ni), or $X_{Co,prec}$, ratio was increasing as indicated in (a-g)

UV/Vis Absorption Spectra (series A1-A4) and Powder X-ray Diffractograms (series A3-A4) of the Co_xNi_{1-x} Nanoparticles

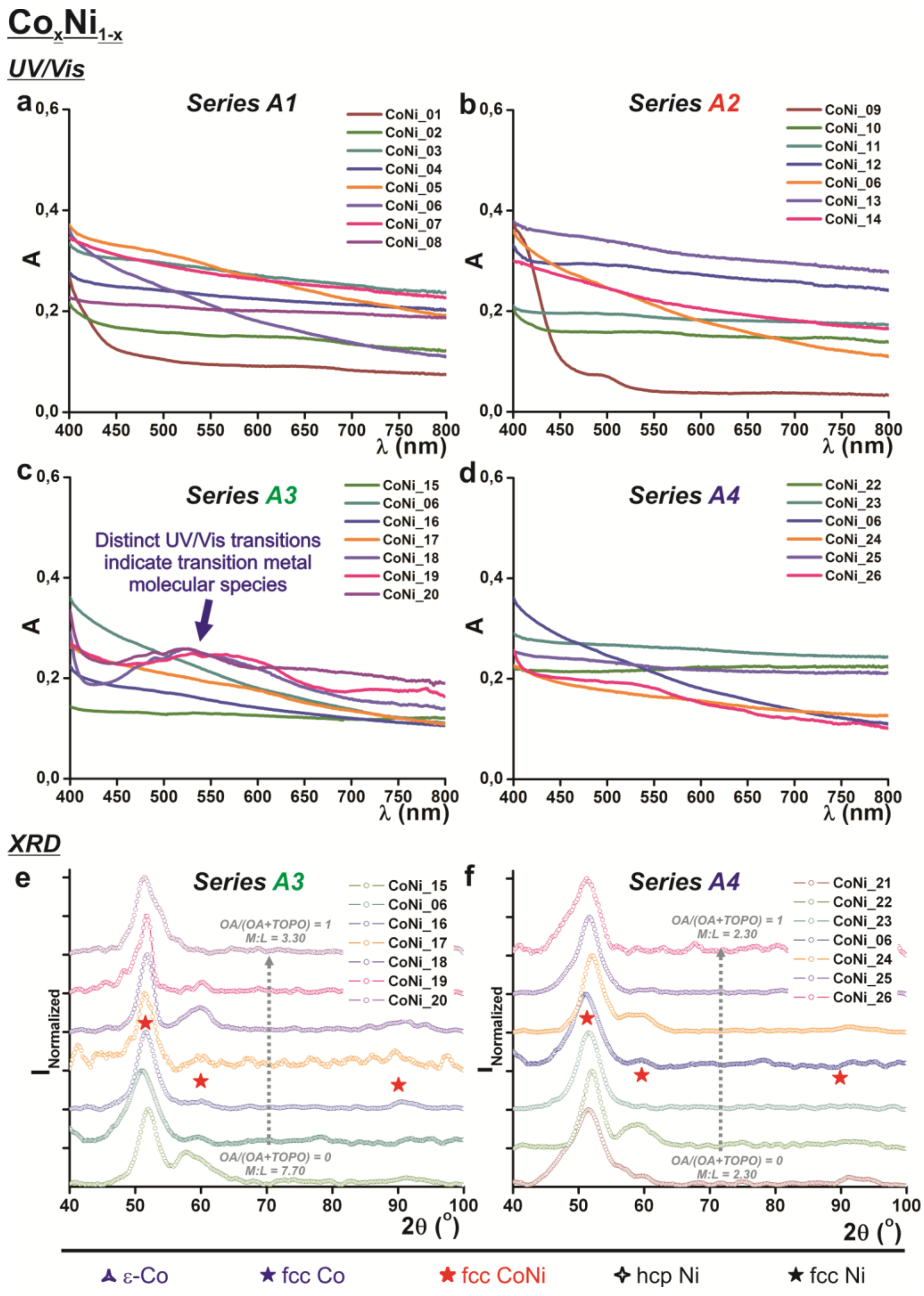

Fig. S6. UV/Vis absorption spectra and powder X-ray diffractograms of raw Co_xNi_{1-x} synthesis products. UV/Vis spectra of the obtained dispersions in series (a) A1, (b) A2, (c) A3, and (d) A4. Typical transition metal nanoparticle optical behavior is observed, as well as the presence of sharp absorption features indicating the presence of molecular complexes. Powder X-ray diffractograms of the same samples as made in synthesis series (e) A3 and (f) A4. XRD patterns were acquired in an inert nitrogen gas atmosphere

Attempted Synthesis of Fe_xNi_{1-x} Nanoparticles

Attempts to synthesize Fe_xNi_{1-x} nanoparticles through the same procedure as described in the Experimental Section of the main text were made by using x mol OA = 0.516*y mol Ni + 0.75*z mol Fe and x mol TOPO = 0.217*y mol Ni + 0*z mol Fe.

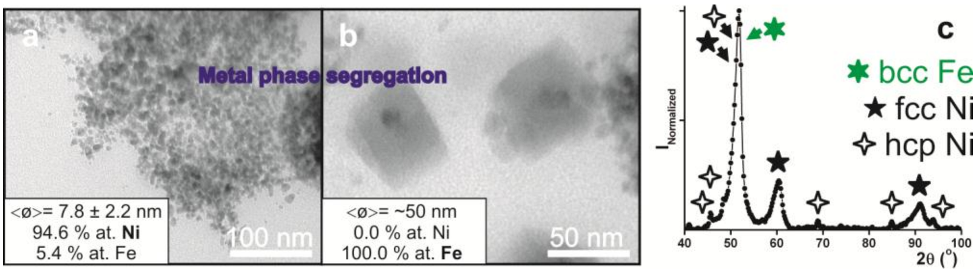

Fig. S7. Characterization of products in attempted Fe_xNi_{1-x} synthesis. Ni nanoparticles of circa 8 nm (a) and pure Fe cubes of circa 50 nm (b) were observed in TEM images. (c) An XRD pattern of the raw synthesis batch revealed both bcc Fe crystallinity, and fcc and hcp Ni crystallinity

Transmission Electron Micrographs of Co_xFe_{1-x} Nanoparticles Made in Series A6

In Fig. S8a-e the synthesis products of series A6 are shown. In summary, all described Co_xFe_{1-x} nanoparticle syntheses in this work yielded essentially spherical nanoparticles.

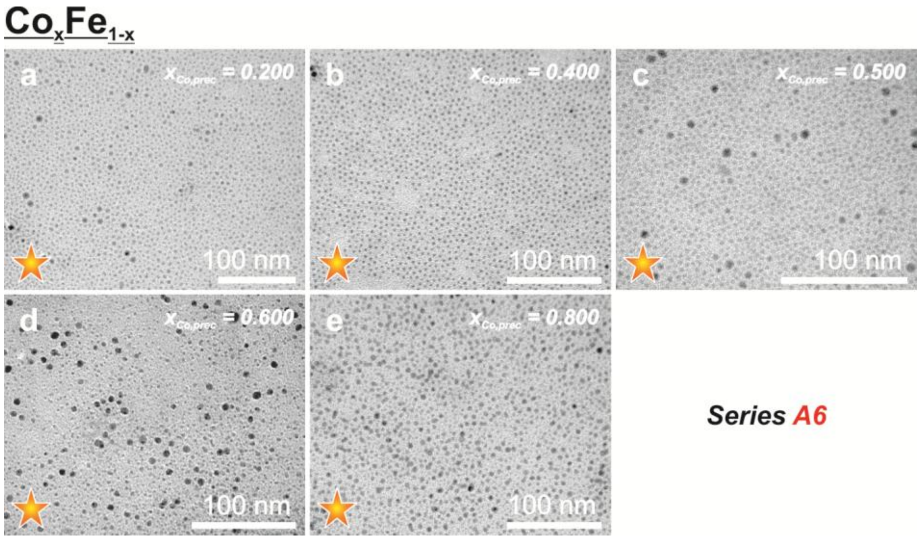

Fig. S8. TEM images of Co_xFe_{1-x} alloy nanoparticles as prepared in synthesis series A6.. In synthesis series A6 a fixed M:L ratio of 1.95, a fixed OA/(OA+TOPO) ratio of 0.90, and varying Co/(Co+Fe) ratios were used, as shown in (a-e). Orange stars indicate syntheses that resulted in 4 to 10 nm spherical Co_xFe_{1-x} nanoparticles

UV/Vis Absorption Spectra and Powder X-ray Diffractograms of the Co_xFe_{1-x} Nanoparticles Synthesized in Series A5-A7

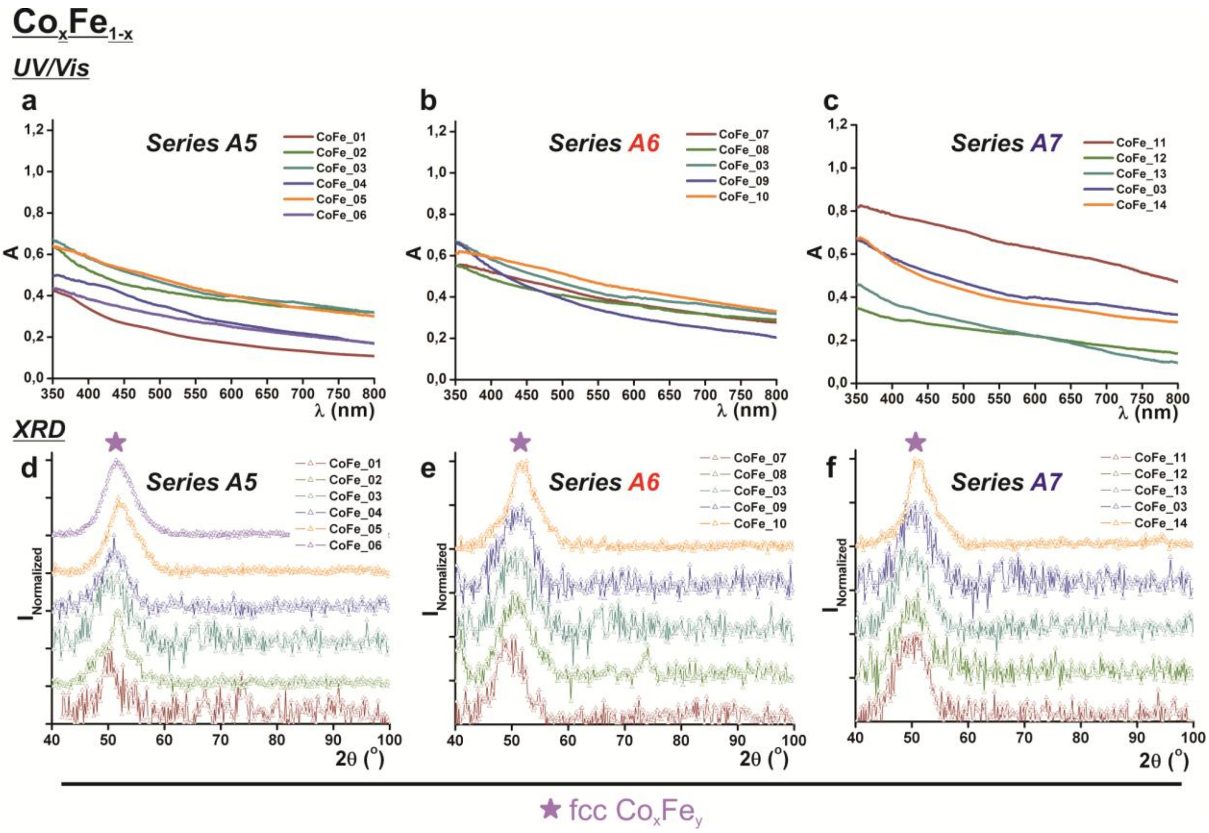

Fig. S9. UV/Vis absorption spectra and powder X-ray diffractograms of raw Co_xFe_{1-x} synthesis products. UV/Vis spectra of the obtained dispersions in series (a) A5, (b) A6, and (c) A7. No specific absorption features were observed suggesting good metal precursor-to-nanoparticle conversion. Powder X-ray diffractograms of the same samples as made in synthesis series (d) A5, (e) A6, and (f) A7. XRD patterns were acquired in an inert nitrogen gas atmosphere
